# Why intention-to-treat matters: preserving the benefits of randomization

**DOI:** 10.36416/1806-3756/e20260304

**Published:** 2026-08-13

**Authors:** Juliana Carvalho Ferreira, Suzana Tanni

**Affiliations:** 1. Methods in Epidemiologic, Clinical, and Operations Research-MECOR-program, American Thoracic Society/Asociación Latinoamericana del Tórax, Montevideo, Uruguay.; 2. Divisão de Pneumologia, Instituto do Coração, Hospital das Clínicas Faculdade de Medicina, Universidade de São Paulo, São Paulo (SP) Brasil.; 3. Departamento de Medicina Interna, Área de Pneumologia, Faculdade de Medicina de Botucatu, Universidade Estadual Paulista - UNESP - Botucatu (SP) Brasil.

## PRACTICAL SCENARIO

The Brazilian Research in Intensive Care Network (BRICNet) designed a randomized controlled trial (RCT) to compare the efficacy of two noninvasive respiratory support strategies-high-flow nasal oxygen (HFNO) and noninvasive ventilation (NIV)-to avoid intubation or death among patients with acute respiratory failure.^1^ After randomization, some patients assigned to HFNO clinically worsen, and their clinicians opted to apply NIV to avoid intubation, prioritizing patients’ safety. Similarly, some patients allocated to NIV were unable to tolerate it and/or failed to improve and were switched to HFNO. In total, 117 of 1,766 randomized patients (6.6%) crossed over to an intervention different from the one they were randomized to. When the trial was completed, investigators faced an important question: should these patients be analyzed according to the treatment they actually received, or according to the group to which they were originally randomized?

## WHAT IS INTENTION-TO-TREAT?

The intention-to-treat (ITT) principle states that participants should be analyzed according to their original treatment allocation, regardless of crossover, nonadherence, treatment discontinuation, or other protocol deviations. In other words, how is the crude value of the intervention?

At first glance, this approach may seem incorrect. After all, if a patient assigned to one intervention ultimately received the other treatment, why not analyze this patient according to the treatment actually received, an approach called per-protocol analysis?

## WHY DOES INTENTION-TO-TREAT MATTER?

The reason is that the main purpose of an RCT is not simply to compare treatments, but to compare groups created through randomization. And randomization is much more than a method for assigning treatments; it is a strategy designed to create comparable groups by balancing both known and unknown confounding factors between study arms.^2^ It ensures that differences in outcomes can be attributed to the intervention rather than to baseline differences in patient characteristics. However, this balance may be compromised if participants are excluded or reassigned after randomization, compromising the premise that the two or more study arms were comparable before receiving the interventions. Therefore, maintaining those groups during the analysis is essential to preserve the validity of this principle-that the two groups are comparable.

## THE PROBLEM WITH PER-PROTOCOL ANALYSIS

Returning to our practical scenario, let us suppose investigators decided that patients who crossed over would be analyzed according to the treatment they actually received. In this case, they are no longer comparing similar groups created by randomization. Instead, they are comparing groups whose composition was influenced by clinical events that occurred after randomization.

The reasons for crossing over in an RCT are often not random. In critical care trials, treatment crossover frequently occurs because patients deteriorate or develop intolerance to an intervention that has side effects. These factors are directly related to prognosis.

If investigators define the groups they are comparing in the analysis based on the treatment they actually received, the protection against confounding provided by randomization is partially lost. As a result, differences in outcomes may reflect differences in patient characteristics or disease severity rather than the effect of the intervention itself.

Similarly, if investigators excluded the patients who crossed over because they did not fully adhere to the assigned treatment, they would interfere with the groups created by randomization. The problem here is that it is likely that many of these patients may have crossed over precisely because they were becoming sicker. Excluding them would result in a study population that is, in reality, a healthier subgroup, potentially making the original intervention appear more effective than it actually is. 

## ITT VERSUS PER-PROTOCOL

ITT and per-protocol analyses address different scientific questions (Chart 1). An ITT analysis estimates the effect of assigning a treatment strategy. It reflects what happens in real-world clinical practice, where nonadherence, crossover, and treatment discontinuation are common. For this reason, ITT is often considered an assessment of effectiveness.

**Chart 1:** Comparison between intention-to-treat and per-protocol approaches.

Characteristic	Intention-to-Treat	Per-Protocol
Population analyzed	All randomized participants, according to groups to which they have been randomized	Participants who adhered to the protocol according to the treatment they actually received
Preserves the benefits of randomization	Yes	No
Main question answered	What is the effect of prescribing this treatment?	What is the effect of the treatment among adherent participants?
Risk of bias	Lower	Higher
Reflects real-world practice	More likely	Less likely
Typical role in RCTs	Primary analysis	Sensitivity analysis

In contrast, a per-protocol analysis includes only participants who adhered to the study protocol and/or tolerated the intervention. This approach attempts to estimate the effect of the intervention under ideal conditions and may provide insights into biological efficacy, but it can overestimate the efficacy of interventions and may provide results that ultimately do not benefit patients in real life. 

Guidelines recommend that RCTs try to minimize crossover as long as patient safety is maintained and that ITT is always used instead of per-protocol analysis. One interesting way of presenting results is performing an ITT-based analysis and also performing a sensitivity analysis using a per-protocol approach.

Investigators should clearly specify which analysis is primary and transparently report protocol deviations and exclusions.

## KEY MESSAGES

- ITT preserves the comparability created by randomization and should be the primary analysis in RCTs.
- Analyzing patients according to the treatment that they actually received may introduce bias, because crossover, protocol deviation, and nonadherence are often related to prognosis.
- ITT and per-protocol analyses answer different questions: effectiveness in real-world practice versus efficacy under ideal conditions.
- Protocol deviations, crossover, and exclusions should be minimized, transparently reported, and explored through sensitivity analyses.

## References

[1] RENOVATE Investigators and the BRICNet AuthorsMaia IS.Kawano-Dourado L.Tramujas L.de Oliveira NE.Souza RN (2025). High-Flow Nasal Oxygen vs Noninvasive Ventilation in Patients With Acute Respiratory Failure The RENOVATE Randomized Clinical Trial. JAMA.

[2] Ferreira JC, Patino CM (2016). Randomization beyond tossing a coin. J Bras Pneumol.

